# Mutation in the Hair Cell Specific Gene* POU4F3* Is a Common Cause for Autosomal Dominant Nonsyndromic Hearing Loss in Chinese Hans

**DOI:** 10.1155/2016/9890827

**Published:** 2016-12-08

**Authors:** Longxia He, Xiuhong Pang, Penghui Chen, Hao Wu, Tao Yang

**Affiliations:** ^1^Department of Otolaryngology-Head and Neck Surgery, Xinhua Hospital, Shanghai Jiaotong University School of Medicine, Shanghai, China; ^2^Ear Institute, Shanghai Jiaotong University School of Medicine, Shanghai, China; ^3^Shanghai Key Laboratory of Translational Medicine on Ear and Nose Diseases, Shanghai, China; ^4^Department of Otorhinolaryngology-Head and Neck Surgery, Taizhou People's Hospital, Jiangsu Province, China; ^5^Department of Otorhinolaryngology-Head and Neck Surgery, Shanghai Ninth People's Hospital, Shanghai Jiaotong University School of Medicine, Shanghai, China

## Abstract

Autosomal dominant nonsyndromic hearing loss (ADNSHL) is extremely heterogeneous. So far the genetic etiological contribution of the gene* POU4F3* associated with ADNSHL has been rarely reported. In our previous study, a c.603_604delGG mutation in the hair cell specific gene* POU4F3* has been identified as the pathogenic cause in one of the seven Chinese Han ADNSHL families. In the present study, we performed targeted next-generation sequencing of 144 known deafness genes in another nine Chinese Han ADNSHL families and identified two more novel mutations in* POU4F3*, p.Leu311Pro and c.120+1G>C, as the pathogenic cause. Clinical characterization of the affected individuals in these three families showed that the three* POU4F3* mutations may lead to progressive hearing loss with variable ages of onset and degrees of severity. Our results suggested that mutations in* POU4F3* are a relatively common cause (3/16) for ADNSHL in Chinese Hans, which should be routinely screened in such cases during genetic testing.

## 1. Introduction

Hearing loss is one of the most common sensorineural defects, which may result from a great variety of genetic and environmental factors. Based on the inheritance patterns, the genetic hearing loss can be classified into autosomal recessive, autosomal dominant, and X-linked/mitochondrial, accounting for approximately 80%, 15%, and less than 5% of nonsyndromic hearing loss, respectively [1]. Both autosomal dominant (ADNSHL) and autosomal recessive (ARNSHL) nonsyndromic hearing loss have an extremely high degree of heterogeneity. For the former, 35 causative genes and over 60 loci have been reported for ADNSHL (The hereditary Hearing Loss Homepage, http://hereditaryhearingloss.org/). So far, mutations in most ADNSHL genes were reported based on studies of the individual cases or families. The etiological contribution of the ADNSHL gene* POU4F3* has been rarely studied. In recent years, however, the development of next-generation sequencing (NGS) has complemented the traditional Sanger sequencing method and made it possible to screen all deafness-associated genes in a high throughout manner [2–4].

Mutations in* POU4F3* have been reported to lead to ADNSHL named as DFNA15 [5]. This gene encodes a 338 amino acids' POU family transcription factor with two conserved DNA-binding domains (the POU-specific domain and the POU homeodomain, amino acids 179−256 and 274−333, resp.). In mouse inner ear, Pou4f3 is strongly expressed in both inner and outer hair cells [6–9]. It activates the transcription of a downstream target gene* Gfi1*, whose expression is required for the maintenance of the outer hair cells [10]. To date, only eleven different mutations in* POU4F3* have been reported in Israeli Jewish, Dutch, Korean, Japanese, and Chinese ADNSHL families [5, 11–17]. The hearing loss caused by* POU4F3* mutations was highly variable in the age of onset, the progression course, and the severity of hearing impairment.

In the previous (*n* = 7) and the present (*n* = 9) studies [18], we preformed targeted NGS of known deafness genes in 16 Chinese Han ADNSHL families and identified novel mutations in* POU4F3* as the pathogenic cause in three of them. Characterization of the hearing phenotype was performed in the affected family members of these three families in the present study. Our results expanded the mutation spectrum of DFNA15 and suggested that mutations in* POU4F3* are a relatively common cause for ADNSHL in Chinese Hans.

## 2. Materials and Methods

### 2.1. Subjects

**Figure 1:**
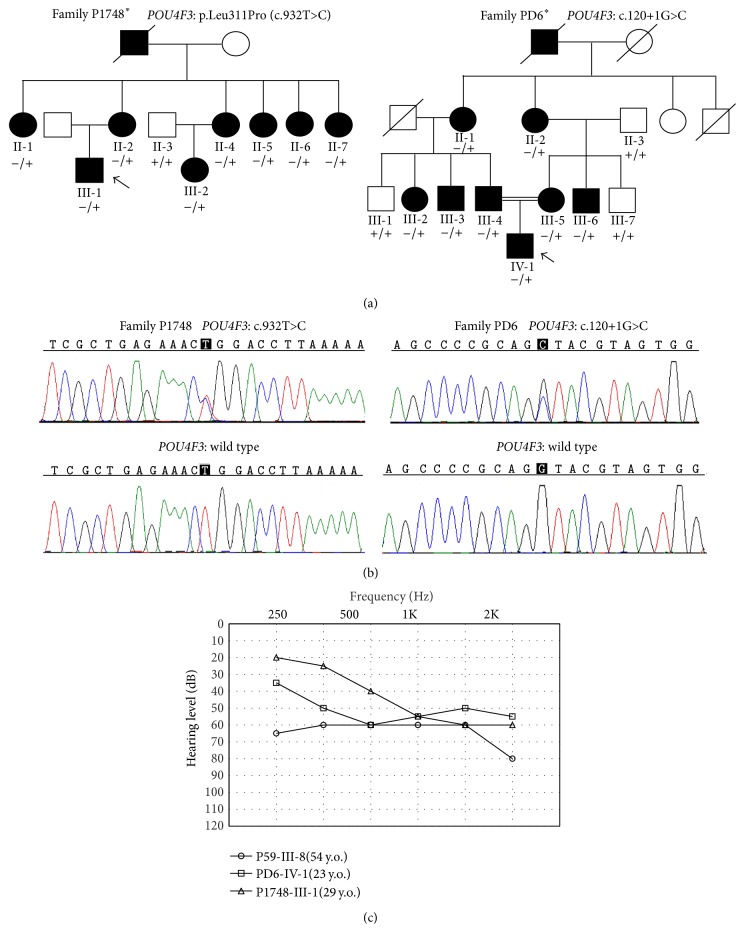
*POU4F3* mutations identified in the Chinese Han ADNSHL families. (a) Pedigrees and genotypes of the families with* POU4F3* mutations. Probands were pointed by arrows. − and + indicate the mutant and wild type alleles, respectively. Asterisks indicate the families with* POU4F3* mutations identified in the present study. (b) Chromatograms showing the c.932T>C (p.Leu311Pro) and the c.120+1G>C mutations in* POU4F3*. (c) Audiograms of the probands of the three families.

Probands of sixteen Chinese Han families segregating ADNSHL were recruited through Xinhua Hospital, Shanghai, China. The pedigrees of the families were shown in Figure 1(a) and Supplementary Figure S1 in Supplementary Material available online at http://dx.doi.org/10.1155/2016/9890827. For Families P1748, PD6, and P59 in which mutations in* POU4F3* were identified, 8, 10, and 13 additional family members were subsequently recruited, respectively. Informed consent was obtained from all subjects. This study was approved by the Ethics Committee of Xinhua Hospital, Shanghai Jiaotong University School of Medicine, Shanghai, China.

### 2.2. Clinical Evaluations

A detailed physical and medical history examination was performed in all probands of the ADNSHL families. The hearing levels were measured by pure tone audiometry (PTA) and shown as the average thresholds of 0.5, 1, 2, and 4 kHz from the better ear. The hearing levels were classified as normal (<20 dB), mild (20–40 dB), moderate (41–70 dB), severe (71–90 dB), and profound (>90 dB).

### 2.3. Mutation Analysis

Targeted NGS of 144 known deafness genes (see complete list in Supplementary Table S1) was performed using the MyGenotics gene enrichment system (MyGenotics, Boston, MD, USA) and the Illumina HiSeq 2000 sequencer (Illumina, San Diego, CA, USA) as previously described [18]. The raw data were first analyzed to filter out the low quality reads. NCBI37/hg19 assembly was used as the reference sequences. Nonsynonymous, on-target variants with maximum minor allele frequency (MAF) less than 0.001 in public databases NHLBI Exome Sequencing Project (ESP, http://evs.gs.washington.edu/EVS/) and the Exome Aggregation Consortium (ExAC, http://exac.broadinstitute.org/) were considered as the candidate pathogenic mutations in compliance with the ADNSHL inheritance. The pathogenicity of the candidate variants was predicted by computational programs Mutation Taster (http://www.mutationtaster.org/), PolyPhen-2 (http://genetics.bwh.harvard.edu/pph2/), PROVEAN, and SIFT (http://sift.jcvi.org/, cutoff scores set at −2.5 and 0.05, resp.). Cosegregation of the pathogenic mutations and the hearing phenotype was verified in members of Families P1748 and PD6 by Sanger sequencing.

## 3. Results

### 3.1. *POU4F3* Mutations Identified in the ADNSHL Families

**Figure 2:**
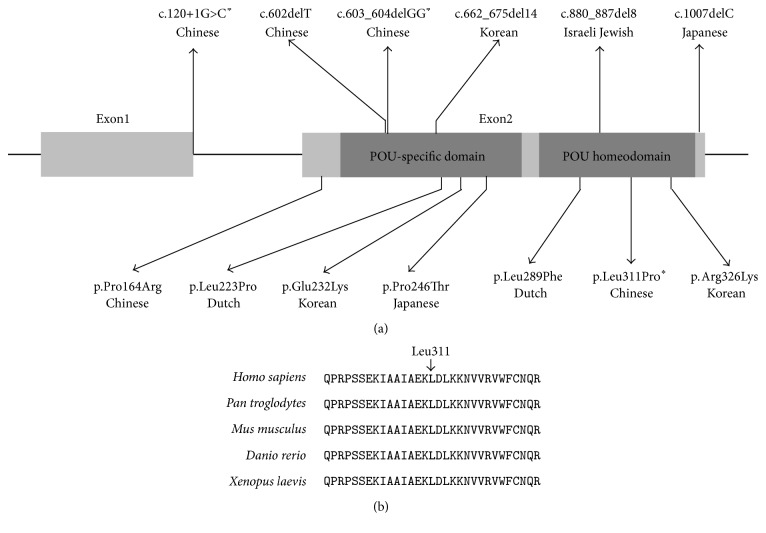
Summary and conservation of the* POU4F3* mutations. (a) Schematic illustration of the thirteen reported* POU4F3* mutations associated with DFNA15. Asterisks indicated the mutations reported in the present study. (b) Multispecies sequence alignment showing the evolutionary conserved amino acid Leu311.

In our previous studies of 7 Chinese Han ADNSHL families, a c.603_604delGG (p.Leu201fs^*∗*^12) mutation in* POU4F3* has been identified by targeted NGS as the pathogenic cause in Family P59 [18]. In the present study, we performed a comprehensive mutation screening in probands of another nine Chinese Han ADNSHL families (marked in asterisks in Figure 1(a) and Supplementary Figure S1) by targeted NGS of 144 known deafness genes. Interestingly, two novel heterozygous variants p.Leu311Pro (c.932T>C) and c.120+1G>C in* POU4F3* were identified as the candidate pathogenic variants in probands P1748-III-1 and PD6-IV-1, respectively (Table 1), along with a heterozygous* TECTA* p.Val1830Met and a heterozygous* TMC1* p.Ser697X variant in proband P1748-1 (Table 1). Sanger sequencing in the rest of the family members confirmed that p.Leu311Pro and c.120+1G>C were the only two candidate variants segregating with the hearing phenotype in Families P1748 and PD6 (Figures 1(a) and 1(b)), while the* TECTA* p.Val1830Met and the* TMC1* p.Ser697X variants were not seen in any other affected family members of P1748. The p.Leu311Pro (c.932T>C) and c.120+1G>C in* POU4F3* were not reported in previous studies, not present in the NHLBI ESP (*n* = 6503) and ExAC (*n* = 60706) database, and not seen in 300 Chinese Han normal hearing controls. The c.120+1G>C mutation was predicted to abolish the 5′ splice site of introns 1 of* POU4F3* (Figure 2(a)). The p.Leu311Pro mutation substituted a conserved amino acid Leu311 (Figure 2(b)) and was predicted to be deleterious by computational programs Mutation Taster, PolyPhen-2, PROVEAN, and SIFT (Table 1).

### 3.2. Clinical Characteristics of the Three ADNSHL Families with* POU4F3* Mutations

Based on the audiograms and the self-description of the affected individuals with c.603_604delGG, p.Leu311Pro, and c.120+1G>C mutations in* POU4F3*, the hearing loss associated with the* POU4F3* mutations was typically progressive with considerable variability in ages of onset and degree of severity both interfamilially and intrafamilially. In Family P1748 with the p.Leu311Pro mutation, proband III-1 had notable hearing loss since age 10 years. The hearing loss affected high frequency most and gradually progressed to moderate hearing loss at age 29 years (Figure 1(c)). The affected individuals in the second generation of Family P1748 had hearing loss at the onset age of 10 years to 20 years and all eventually progressed to profound after age 50 years. Interestingly, all six female patients in the second generation had significantly decreased hearing levels after giving birth to their children. In Family PD6 with the c.120+1G>C mutation, proband IV-1 had congenital, moderate hearing loss with a relatively “flat” audiometric profile at age 23 years affecting all frequencies (Figure 1(c)). Other affected individuals in this family demonstrated progressive, moderate-to-profound deafness depending on their ages. The ages of onset ranged from congenital to 40 years. In Family P59 with the c.603_604delGG mutation, the proband III-8 had only moderate hearing loss at 54 years of age (Figure 1(c)) and all affected individuals in this family had a rather late age of onset around 40s. For other auditory symptoms, tinnitus has been complained by proband P1748-III-1. No vestibular dysfunction was shown in any affected individuals.

## 4. Discussion

Combined with our present and previous studies, we identified mutations in* POU4F3* as the pathogenic cause of deafness in 3 of the 16 (18%) Chinese Han ADNSHL families, suggesting that it is a relatively common cause for ADNSHL in Chinese Hans. Consistently, seven of the ten previously reported* POU4F3* mutations from other research groups were also from the East Asians (three in Korean, two in Japanese, and two in Chinese, Figure 2(a)), suggesting that this gene should be routinely screened in ADNSHL cases of East Asian descent. In contrast, only three (one in Israeli Jewish and two in Dutch, Figure 2(a))* POU4F3* mutations were previously reported from regions other than East Asia. The distinguished mutation spectrum among different ethnical groups has also been reported for other deafness genes such as* SLC26A4*, in which case biallelic* SLC26A4* mutations can be identified in 88.4% of deaf patients with nonsyndromic EVA in Chinese but only 15% in Caucasians [19, 20].

Our study also expanded the mutation spectrum of* POU4F3*. Figure 2(a) summarized the type, position, and associated ethnicity of the thirteen* POU4F3* mutations reported to date. Four of them, including the c.603_604delGG (p.Leu201fs^*∗*^12) mutation reported in our previous study, were truncating mutations that were predicted to lead to prematurely stopped protein product or nonsense-mediated decay of the mRNA, while another seven* POU4F3* mutations, including the p.Leu311Pro mutation reported in the present study, were missense mutations leading to single amino acid substitutions. Notably, these twelve mutations were all located within or close to the POU-specific domain or the POU homeodomain, the two conserved DNA-binding domains of POU4F3 encoded in exon 2. On the contrary, the c.120+1G>C mutation identified in the present study is the only reported mutation outside exon 2 of* POU4F3*.

Consistent with previous reports [5, 11, 13–16], the* POU4F3* mutations identified in the present study were associated with progressive hearing loss with considerable variability in the ages of onset and the degrees of severity, and this variable hearing phenotype can be seen both interfamilially and intrafamilially. Apparently no simple genotype-phenotype correlation can be drawn based on the position or the truncating/nontruncating nature of the* POU4F3* mutations. In a previous study of the* Pou4f3* mutant deaf mice, deficiency of Pou4f3 has been found to result in reduced expression of its hair cell specific downstream target* Gfi1*, which was suggested as the direct cause of the outer hair cell degeneration in the* Pou4f3* mutant mice [10]. In future studies, therefore, it will be interesting to correlate the presumably reduced levels of the* Gfi1* transcription with different* POU4F3* mutations and the severity of the associated hearing loss.

## 5. Conclusions

Mutations in* POU4F3* are a relatively common cause for ADNSHL in Chinese Hans. The hearing loss associated with* POU4F3* mutations has considerable variability in the ages of onset and the degrees of severity.

## Supplementary Material

The Supplementary Material includes Supplementary Table S1 - 144 genes targeted for the next-generation sequencing and Supplementary Figure S1 - Family P59 and the thirteen additional Chinese Han ADNSHL families without POU4F3 mutations identified by targeted NGS.

## Figures and Tables

**Table 1:** Candidate pathogenic mutations identified in probands of Families P1748 and PD6 by targeted NGS.

Proband	Gene (reference sequence)	Mutation	MAF (ExAC)	MAF (NHLBI ESP)	Mutation Taster	PROVEAN (score)	SIFT (score)	PolyPhen-2 (HumVar score)	Intrafamilial phenotype cosegregation
P1748-III-1	POU4F3 (NM_002700)	p.Leu311Pro (c.932T>C)	0	0	Disease causing	Deleterious (−3.63)	Damaging (0)	Probably damaging (1)	Yes
	TECTA (NM_005422)	p.Val1830Met (c.5488G>A)	0.0003871	0	Disease causing	Neutral (−0.83)	Damaging (0.008)	Probably damaging (0.969)	No
	TMC1 (NM_138691)	p.Ser697X (c.2090C>G)	0	0	Disease causing	Deleterious (−10.26)	—	—	No

PD6-IV-1	POU4F3 (NM_002700)	c.120+1G>C	0	0	Disease causing	—	—	—	Yes
