# Supplementary material for: Mutation in the Hair Cell Specific Gene POU4F3 Is a Common Cause for Autosomal Dominant Nonsyndromic Hearing Loss in Chinese Hans
Source: Neural Plast. 2016 Dec 8;2016:9890827. doi: 10.1155/2016/9890827 (PMC5178374; doi:10.1155/2016/9890827)
Supplement: Supplementary file 1 — The Supplementary Material includes Supplementary Table S1 - 144 genes targeted for the next-generation sequencing and Supplementary Figure S1 - Family P59 and the thirteen additional Chinese Han ADNSHL families without POU4F3 mutations identified by targeted NGS. [file 9890827.f1.pdf]

Mutations in the hair cell specific gene *POU4F3* is a common cause for autosomal dominant non-syndromic hearing loss in Chinese Hans

Longxia He[§1,2,3], Xiuhong Pang[§4], Penghui Chen[§1,2,3], Hao Wu[*1,2,3,5] and Tao Yang[*1,2,3]

[1]Department of Otolaryngology–Head and Neck Surgery, Xinhua Hospital, Shanghai Jiaotong University School of Medicine, Shanghai, China

[2]Ear Institute, Shanghai Jiaotong University School of Medicine, Shanghai, China

[3]Shanghai Key Laboratory of Translational Medicine on Ear and Nose Diseases, Shanghai, China

[4]Department of Otorhinolaryngology-Head and Neck Surgery, Taizhou People's Hospital, Jiangsu Province, China

[5]Department of Otorhinolaryngology-Head and Neck Surgery, Shanghai Ninth People's Hospital, Shanghai Jiaotong University School of Medicine, Shanghai, China

[§]These authors contributed equally to this work

***Corresponding Authors:**

Tao Yang, Ear Institute, Shanghai Jiaotong University School of Medicine, Shanghai, China. Email: yangtfxl@sina.com

Hao Wu, Department of Otolaryngology-Head & Neck Surgery, Shanghai Ninth People's Hospital, Shanghai Jiaotong University School of Medicine, Shanghai, China. Email: wuhao622@sina.cn

Supplementary Table S1. The 144 genes targeted for the next-generation sequencing for deafness.

ACTG1	COL9A1	FGF8	HSD17B4	MYO15A	PROKR2	TBC1D24
ALX3	COL9A2	FGFR1	IL13	MYO1A	PRPS1	TCOF1
BSND	COMT2	FGFR3	ILDR1	MYO3A	PTPRQ	TECTA
CABP2	CRYM	FLNA	KARS	MYO6	RDX	TIMM8A
CCDC50	DFNA5	FOXI1	KCNE1	MYO7A	RPGR	TJP2
CDH23	DFNB31	FREM1	KCNJ10	NDP	SALL1	TMC1
CEACAM 16	DFNB59	GATA3	KCNQ1	NF2	SALL4	TMIE
CHD7	DIABLO	GIPC3	KCNQ4	OTOA	SANS	TMPRSS3
CIB2	DIAPH1	GJB2	KRT9	OTOF	SEC23A	TNC
CLDN14	DIAPH3	GJB3	LAMA3	P2RX2	SEMA3E	TPRN
CLPP	DSPP	GJB6	LARS2	PABPN1	SERPINB 6	TRIOBP
CLRN1	ECM1	GPR98	LHFPL5	PAX3	SIX1	TRMU
COCH	EDN3	GPSM2	LOXHD1	PCDH15	SIX5	TSPEAR
COL11A1	EDNRB	GRHL2	LRTOMT	PDZD7	SLC17A8	USH1C
COL11A2	ELMOD3	GRXCR1	MARVEL D2	PNPT1	SLC26A4	USH1G
COL2A1	ESPN	HARS	MIR96	POLR1C	SLC26A5	USH2A
COL4A3	ESRRB	HARS2	MITF	POLR1D	SMPX	WFS1
COL4A4	EYA1	HGF	MSRB3	POU3F4	SNAI2	WHRN
COL4A5	EYA4	HMX1	MYH14	POU4F3	SOX10	RNR1
COL4A6	FGF3	HOXA2	MYH9	PROK2	STRC	TS1
CO1	miR-96	miR-182	miR-183			

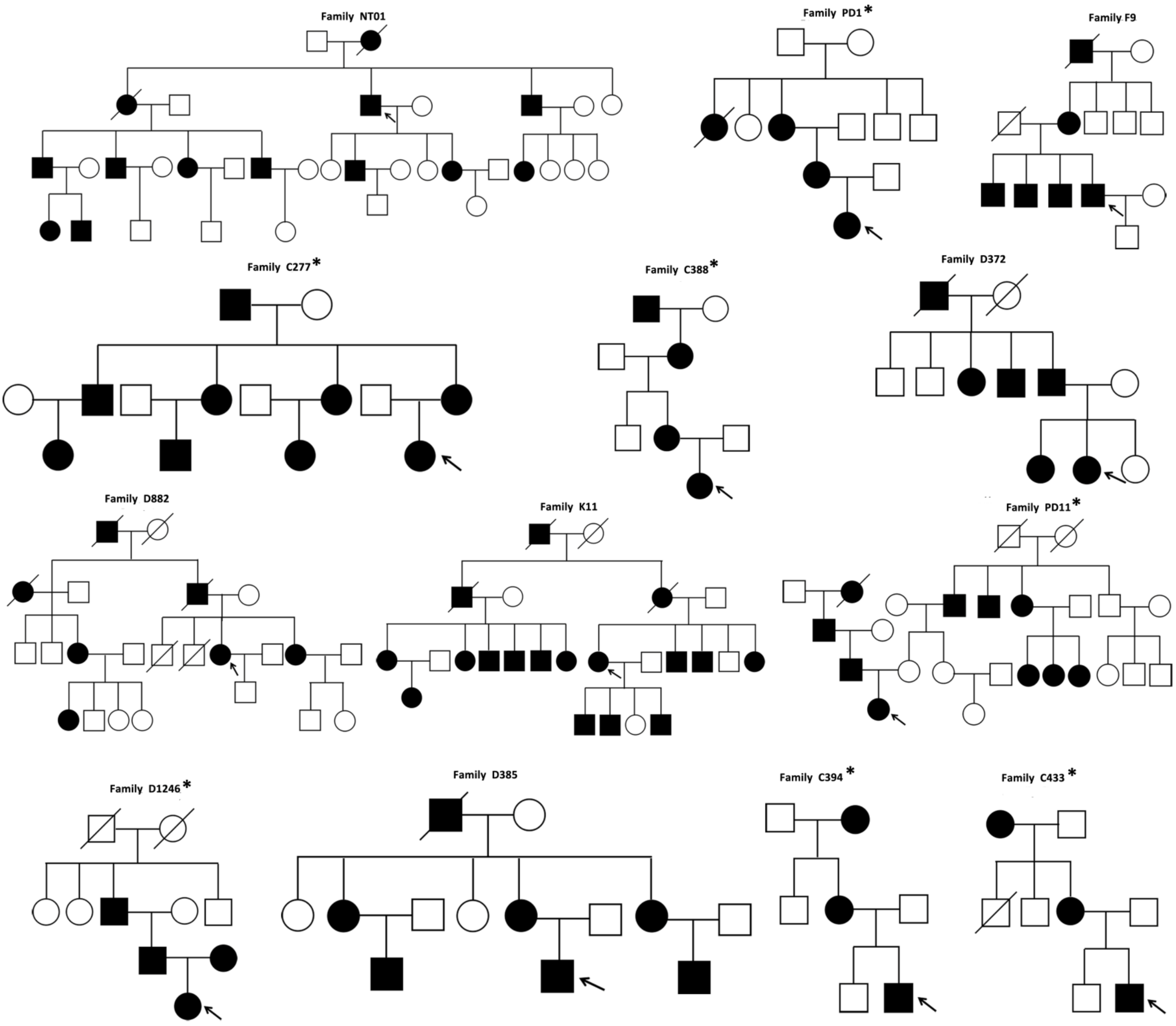

Supplementary Figure S1. The thirteen additional Chinese Han ADNSHL families without *POU4F3* mutations identified by targeted NGS. Asterisks indicate the families screened in the present study. Probands were pointed by arrows.
